# Oral frailty: a concept analysis

**DOI:** 10.1186/s12903-024-04376-6

**Published:** 2024-05-22

**Authors:** Huimin Zhao, Bei Wu, Yuqiu Zhou, Zhilan Yang, Hua Zhao, Ziwei Tian, Manhong Jiang, Deqin Huang

**Affiliations:** 1 College of Nursing, Shanxi University of Chinese Medicine, Jinzhong, 030619 China; 2 Rory Meyers College of Nursing and NYU Aging Incubator, New York University, New York, NY 10012 USA; 3 Department of Nursing, Harbin Medical University, Harbin, 150081 China; 4 School of Nursing, Hangzhou Normal University, Hangzhou, 311121 China

**Keywords:** Oral frailty, Concept analysis, Review

## Abstract

**Background:**

Oral frailty has become a worldwide problem among older adults. Although researchers have conducted various studies on oral frailty, its definition remains controversial.

**Purpose:**

To clarify the concept of oral frailty.

**Methods:**

Online databases PubMed, Web of Science, CINAHL, Cochrane Library, ProQuest, China National Knowledge Infrastructure (CNKI), China Science and Technology Journal Database (VIP), and Wanfang database were searched from inception to September 20, 2023. The reference lists of relevant studies were searched manually. Eligible articles, theses, and books were analyzed using Walker & Avant’s concept analysis model.

**Results:**

The attributes of oral frailty were abnormal oral structure and/or decline in multi-faceted oral function and coexisting decline in physical, cognitive and social functions. Its antecedents were aging, social frailty, and severe periodontitis, whereas its consequences were decline in physical health and mental health, social withdrawal, lower quality of life and systemic frailty.

**Conclusion:**

Oral frailty could result in worse conditions among older adults physically, psychologically and socially. Tools based on the concept analysis need to be developed to comprehensively assess oral frailty.

**Supplementary Information:**

The online version contains supplementary material available at 10.1186/s12903-024-04376-6.

## Introduction

The global population is rapidly aging, and the poor oral health conditions and problems of older adults have become a serious problem that could not be ignored. Studies found that older adults suffered from oral health problems, such as missing teeth, worse oral hygiene, subjective difficulties in eating and swallowing, etc [1]. These oral health problems lead to impaired physical health, cognitive function, decreased social function, reduced quality of life and even increased risk of death in older adults [2–4].

“Oral frailty” was first proposed by the Japanese Society of Gerontology in 2013, which was defined as an age-related decrease in oral function [5]. Since then, the research on oral frailty has been increased, including its status quo, assessment tools, and intervention strategies [6], which provided evidence to cope with oral frailty among older adults. However, researchers have defined oral frailty in different ways. Some researchers indicated that oral frailty was an early stage of oral hypofunction [7], however, others considered these two concepts were totally different: oral frailty is a disease concept, and oral hypofunction is a condition [8]. Hihara et al. assumed that oral frailty is an early manifestation of physical frailty [9].

The Japan Dental Association proposed a more comprehensive definition of oral frailty, which referred to a series of phenomena and processes that lead to changes in various oral conditions (such as number of teeth, oral hygiene, and oral function) associated with aging and accompanied by decreased interest in oral health, reduced physical and mental reserve capacity, and an increase in oral frailty resulting in eating dysfunction [10]. This concept defined oral frailty from the aspects of its clinical manifestations, causes, and consequences. However, new research evidence on oral frailty has been produced since then, and the understanding of the concept of oral frailty kept changing. For example, the attributes of oral frailty, defined as oral health changes, did not seem to be very precise; as evidence on the causes and consequences of oral frailty increases, aging is not the only factor that could cause oral frailty. The consequences of oral frailty are also not just limited to increased oral susceptibility. The clarification of oral frailty is needed.

Walker and Avant proposed to clarify a concept from the perspective of reviewing its uses, identifying attributes, antecedents, consequences, empirical referents and constructing cases, which is a reliable and widely-used way to define a concept [11, 12]. This study aimed to clarify the concept of oral frailty, so that the correct application of oral frailty in the field of research, clinical practice and education could be achieved.

## Methods

The methods of Walker & Avant were used to analyze the concept of oral frailty [11, 12]. Online databases were searched from inception to September 20, 2023, and we searched dictionaries as well and hand-searching were conducted.

### Concept analysis method

Walker & Avant’s concept analysis method is a systematic and reliable approach, which is also the most commonly used method for concept analysis in nursing research [11, 12]. We used Walker & Avant’s concept analysis method to identify antecedents, attributes, consequences and empirical referents of the concept of oral frailty as well as constructing cases. The 8-step analysis includes: (1) selecting the concept; (2) determining the purpose of analysis; (3) identifying all uses of the concept; (4) defining attributes; (5) identifying a model case; (6) identifying borderline, related and contrary cases; (7) identifying antecedents and consequences; and (8) identifying the empirical referents of the defining empirical attributes.

### Data sources

Online databases and Dictionaries were searched for the concept analysis. Data sources included the resources described below.

#### Online databases

We systematically searched PubMed, Web of Science, CINAHL, Cochrane Library, and ProQuest using the term “oral frailty”. The search also included several major Chinese databases, including China National Knowledge Infrastructure (CNKI), China Science and Technology Journal Database (VIP), and Wanfang database using the Chinese term “口腔衰弱 (oral frailty)”. In addition, the reference lists of relevant studies were searched by hand.

#### Dictionaries

We searched the Cambridge Dictionary and Merriam-Webster Dictionary online to determine the definition of oral frailty.

### Selection criteria

The inclusion criteria were as follows: literature containing definitions, attributes, causes and outcomes of oral frailty, language in both English and Chinese, books related to oral frailty; qualitative, quantitative, mixed methods or systematic reviews. Exclusion criteria were as follows: repeated reported literature; editorial and conference papers; the full text was not available.

### Data collection and analysis

A systematic search of the literature was completed using standard review processes. Two authors independently applied a two-step study selection procedure to determine if the article accepts [13], and disagreements at each step were resolved through discussion and consultation with a third author.

## Results

**Fig. 1:**
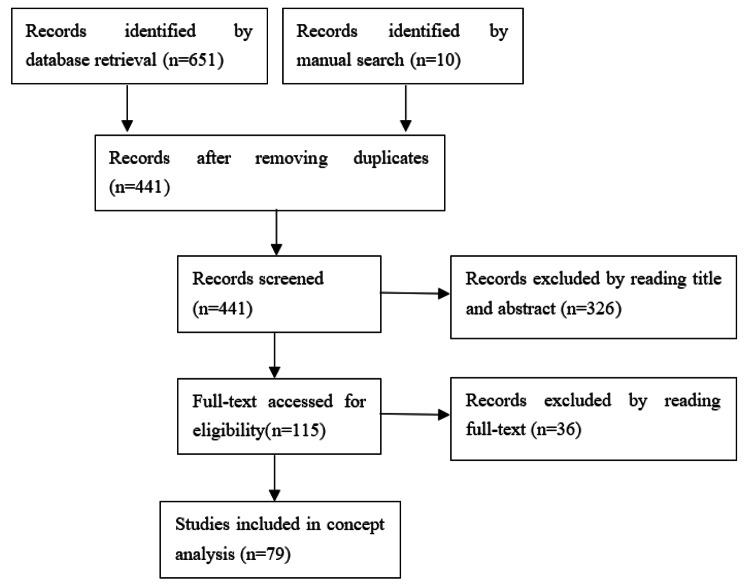
Flow diagram of literature search

651 studies totally were searched in online databases. 10 studies were identified by manual search. After removing duplicates, 441 studies were screened by reading titles and abstracts. Then 326 studies were excluded and 115 studies were accessed for eligibility by reading full-texts. Eventually, 79 studies were included for concept analysis using Walker & Avant’s concept analysis method. The flow diagram of literature search is shown in Fig. 1.

### Uses of the concept

#### Definition of Dictionary

Walker and Avant indicated that identifying all possible uses of the concept was the first step for analyzing the attributes of the concept [11]. There is no word in the Cambridge Dictionary and Merriam-Webster Dictionary for oral frailty. However, the related terms “oral” and “frailty” were identified respectively. “Oral” means “of, taken by, or done to the mouth” and “frailty” means “weakness and lack of health or strength” in Cambridge Dictionary [14, 15]. In Merriam-Webster Dictionary, “oral” means “of, given through, or involving the mouth” and “frailty” refers to “the quality or state of being frail” [16, 17].

#### Use of oral frailty in medicine

The original definition of oral frailty was introduced in Japan in 2013, which means “decline of oral function associated with aging” [18]. Since then, many researchers proposed similar definitions for oral frailty. They indicated that oral frailty is a mild decline in oral function, deterioration of oral function or loss of oral function [7–9, 19–26], and proposed that oral frailty is a precursor or accelerator of general frailty [9, 19, 20, 25].

Apart from describing oral frailty as decline in oral function or loss of oral function, Dibello et al. proposed that the loss of oral function is driven by a set of impairments that worsen oral daily functions—eg, loss of teeth, poor oral hygiene, inadequate dental prostheses, or difficulty in chewing associated with age-related changes in swallowing [27]. Likewise, Minakuchi et al. [28], Hihara et al. [9], and Doi et al. [29] specifically described the manifestations of oral frailty.

A more widely used concept was proposed by the Japan Dental Association [10]. Oral frailty hereby refers to a series of phenomena and processes in which various oral health conditions (such as number of teeth, oral hygiene, and oral function) change with aging, resulting in vulnerability in oral health conditions, accompanied by decreased interest in oral health and the physical and mental reserve capacity [10]. Similarly, Kusunoki et al. defined oral frailty as an age-related gradual loss of oral functions, accompanied by a decline in cognitive and physical functions [30].

Except for the conceptual definition, researchers developed the operational definition of oral frailty as well [31, 32]. As of 2018, Tanaka et al. defined the conceptual definition of oral frailty as the accumulation of poor oral health. They then proposed the operational definition as the co-existence of a poor status in ≥ 3 out of the six measures: the number of natural teeth, chewing ability, articulatory oral motor skill, tongue pressure, subjective difficulties in eating and swallowing [31]. Parisius et al. developed the definition of oral frailty as the age-related functional decline of orofacial structures [33], and proposed that its operational definition should include difficulty eating hard or tough foods, inability to chew all types of foods, decreased ability to swallow solid foods, decreased ability to swallow liquids, overall poor swallowing function, impaired tongue movement, speech or phonatory disorders, and hyposalivation or xerostomia [32]. The different uses of the concept are described in Table 1.

**Table 1:** Summary of the different uses of the concept

Sources	Resource type	Definition	Attributes	Antecedents	Consequences	Population	Empirical referents
Cambridge Dictionary [14, 15]	Dictionary	“Oral” means “of, taken by, or done to the mouth” and “frailty” means “weakness and lack of health or strength” ^(a)^	/	/	/	/	/
Merriam-Webster Dictionary [16, 17]	Dictionary	“Oral” means “of, given through, or involving the mouth” and “frailty” refers to “the quality or state of being frail” ^(a)^	/	/	/	/	/
The Japanese Society of Gerontology, 2013 [18]	Literature review	Decline of oral function associated with aging ^(a)^	A	/	Physical frailty, sarcopenia, severe conditions requiring nursing care, death	Older adults ≥ 65 years old	/
Kera et al., 2017 [21]	Quantitative research	A decline in oral function ^(a)^	A	/	/	Individuals over 65 years of age	The Kihon Checklist
Minakuchi et al., 2018 [28]	Literature review	Frailty that manifests only in the oral cavity with signs or symptoms specified as decreased articulation, slight choking or spillage while eating, and an increased number of unchewable foods ^(a)^	A	/	/	Older adults	/
Naruishi et al., 2018 [22]	Quantitative research	Age-related vulnerable/impaired oral functions including poor oral health ^(a)^	A	/	Aspiration pneumonia	Elderly patients	/
Tanaka et al., 2018 [31]	Quantitative research	The accumulation of poor oral health ^(a)^; The co-existence of a poor status in ≥ 3 out of the six measures: the number of natural teeth, chewing ability, articulatory oral motor skill, tongue pressure, subjective difficulties in eating and swallowing ^(b)^	A	Being older	Physical frailty, sarcopenia, disability, mortality	Community-dwelling elderly individuals (≥ 65 years old)	Oral Frailty Status
Hihara et al., 2019 [9]	Quantitative research	A mild decline in oral function, including symptoms such as the decline in tongue action, spilling foods, and slight choking, which is one of the earliest stages of physical frailty ^(a)^	A, B	Being older	/	Subjects with ages over 65 years	/
Satake et al., 2019 [19]	Quantitative research	The loss of oral function and a precursor or accelerator of general frailty ^(a)^	A, B	Aging	General frailty	Community-dwelling volunteers aged ≥ 60 years	/
The Japan Dental Association, 2019 [10]	Manual	A series of phenomena and processes in which various oral health conditions (such as number of teeth, oral hygiene, and oral function) change with aging, resulting in vulnerability in oral health conditions, accompanied by decreased interest in oral health and the physical and mental reserve capacity ^(a)^	A, B	/	Physical frailty, mortality	Older adults at various types of medical and long-term care sites	/
Hasegawa et al., 2020 [20]	Quantitative research	A slightly impaired oral function and compromised oral hygiene, which are early symptoms of general physical frailty ^(a)^	A, B	Age	Social withdrawal	Rural older adults aged 65 years or older	The Kihon Checklist
Matsuo et al., 2020 [23]	Quantitative research	Poor oral health or oral hypofunction ^(a)^	A	/	/	Young and older adults	/
Nishida et al., 2020 [24]	Quantitative research	A very rapid stage of oral dysfunction ^(a)^	A	/	/	Community-dwelling older adults aged ≥ 65 years	/
Dibello et al., 2021 [27]	Literature review	Age-related gradual loss of oral function, driven by a set of impairments that worsen oral daily functions—eg, loss of teeth, poor oral hygiene, inadequate dental prostheses, or difficulty in chewing associated with age-related changes in swallowing ^(a)^	A	/	/	Older adults > 60 years old	/
Nomura et al., 2021 [25]	Quantitative research	The mild decline in oral function and located at the early and reversible stage of frailty ^(a)^	A, B	/	/	Subjects more than 50 years old	The Oral Frailty Checklist
Minakuchi, 2022 [8]	Literature review	The deterioration of oral function ^(a)^	A	/	/	/	/
Parisius et al., 2022 [33]	Literature review	The age-related functional decline of orofacial structures ^(a)^	A	/	/	Older adults	/
Doi et al., 2023 [29]	Quantitative research	A slight decline in oral function (decreased movement of the tongue to pronounce, spilled food, difficulties in chewing well, etc.) ^(a)^	A	Social frailty	Social withdrawal, disability, mortality	Residents aged 75, 80, 85, and 90 years old	/
Kusunoki et al., 2023 [30]	Quantitative research	An age-related gradual loss of oral functions, accompanied by a decline in cognitive and physical functions ^(a)^	A, B	/	Mortality, physical frailty, systemic frailty, functional disability, poor quality of life	Patients aged ≥ 65 years	The Oral Frailty Index-8 (OFI-8)
Parisius et al., 2023 [32]	Qualitative research	The operational definition includes difficulty eating hard or tough foods, inability to chew all types of foods, decreased ability to swallow solid foods, decreased ability to swallow liquids, overall poor swallowing function, impaired tongue movement, speech or phonatory disorders, and hyposalivation or xerostomia ^(b)^	A	/	/	/	/
Tanaka et al., 2023 [26]	Quantitative research	The overlap of minor declines in dental or oral functions that may increase the risk of adverse health outcomes ^(a)^	A	Age	Physical frailty, physical disability, mortality	Community-dwelling older adults ≥ 65 years old	The Oral Frailty Five-item Checklist (OF-5)

^(a)^ Conceptual definition

^(b)^ Operational definition

A Abnormal oral structure and/or decline in multi-faceted oral function

B Coexisting decline in physical, cognitive and social functions

#### Related terms

##### Oral hypofunction

Researchers early assumed that oral frailty was an early stage of oral hypofunction [28]. Oral frailty is defined as mild decline in oral function, and is reversible in the early stage [7]. However, a study clarified that oral frailty is a separate concept, not its early stage. Oral frailty is a disease concept, and oral hypofunction is a condition [8]. In 2020, the public insurance system in Japan presents oral hypofunction as a disease, a complex decline in oral function, not only due to aging, but also caused by various factors such as diseases and disorders [34].

Oral frailty and oral hypofunction both represent a decrease in oral function [35]. Oral hypofunction is diagnosed based on 7 signs or symptoms: oral hygiene, oral moisture, biting force, tongue and lip motor function, tongue pressure, chewing function, and swallowing function. It could be diagnosed if 3 or more signs or symptoms were met [28]. Oral frailty is diagnosed by tooth count, chewing ability, oral motor skills, tongue pressure, subjective eating and swallowing difficulties [31].

Some researchers consider oral hypofunction as a core component of oral frailty [36]. According to Japan Dental Association, the third level of oral frailty was “oral hypofunction”, other levels of oral frailty were decline of oral health literacy, oral function with slight problem, and oral function disorders [10]. Currently, the relationship between oral hypofunction and oral frailty fails to reach a consensus and further research is needed to explore it.

##### Oral pre-frailty

Oral pre-frailty is an early stage of oral frailty and is defined as reduced function in 1–2 of 6 measures (the number of natural teeth, chewing ability, articulatory oral motor skill, tongue pressure, subjective difficulties in eating and swallowing) [20].

### Attributes

#### Abnormal oral structure and/or decline in multi-faceted oral function

Abnormal oral structure includes atrophy of alveolar bone, reduction in the number of muscle fibers of the masticatory muscle, changes of oral mucosa, wear of the structure and composition of the dental hard tissues, enamel chipping/cracking/fracture, and tooth loss [37, 38]. Tanaka et al. [31] and Iwasaki et al. [39] indicated that the issue of tooth loss was one of the concerns regarding oral frailty. Tanaka et al. specially reported that the number of natural teeth was a measure for oral frailty [31]. Ichikawa et al. used the moisture of oral mucosa as one of the variables evaluating oral frailty [40]. In addition, previous studies showed that the decline of oral structure was included in the definition of oral frailty [33, 41]. Therefore, abnormal oral structure is considered part of the attribute of oral frailty. Except for the number of natural teeth, Tanaka et al. proposed other measures of oral frailty. For example, chewing ability fell into group of decline in oral function [31]. Plus, the currently widely accepted and used operational definition of oral frailty developed by Tanaka et al. proposed that coexisting poor status in 3 or more measures met the criteria of oral frailty [31]. Hiltunen et al. evaluated oral frailty based on six signs: dry mouth, eating mud or soft food, food residue on the oral surface, slurred speech, inability to open mouth during clinical oral examination, and pain during examination [42]. Thus, oral frailty is multi-faceted.

#### Coexisting decline in physical, cognitive, and social functions

Oral frailty is an important part of general frailty [43]. A study showed that physical frailty was closely associated with oral frailty [44]. A cross-sectional study indicated that oral frailty was associated with lower gait speed, shorter stride and step length, wider step width, and longer double support duration, and higher variability of stride length and step length among community-dwelling older adults [45]. A decline in cognitive function is another aspect of oral frailty [27]. Studies indicated decline in social function interacted with oral frailty [20, 46], hence it belongs to the coexisting conditions of oral frailty.

### Constructing cases

#### Model case

Grandpa Li, 70 years old, retired, lives in a nursing home now. He was diagnosed with mild cognitive impairment and frailty *(coexisting decline in physical and cognitive functions)*. When doctor examined his oral conditions, the doctor found he has fewer natural teeth (*abnormal oral structure*). Grandpa Li told the doctor, he felt difficult chewing and swallowing solid food (*decline in multi-faceted oral function*). The loss of natural teeth and difficulty in eating food made him feel frustrated, and he seldom communicated with other people in nursing home (*coexisting decline in social function*). He thought he was ugly with few teeth, especially when he talked with his family *(coexisting decline in social function)*. Plus, he told the doctor he often forgot to brush teeth and this might be the reason why he has few teeth now (*coexisting decline in cognitive function*). Another reason, he said, “I’m getting old and I’m not as fit as I used to be, so my teeth aren’t working”.

According to Walker and Avant, a model case means the case includes all the attributes of a concept [11]. This is a model case of oral frailty, because it includes both attributes of oral frailty.

#### Contrary case

Mrs. Wang, 80 years old, lives in a community with her family. She maintains a good state of physical and mental health. Her number of teeth is over 20. Her chewing ability, articulatory oral motor skill, and tongue pressure is normal after oral examination. When doctor asked if she had difficulties in eating and swallowing, she said “no”. She always takes the initiative to talk with other people and her good mood often influences people around her.

As for Walker and Avant, a contrary case refers to a case does not include any attributes of a concept [11]. Mrs. Wang does not have decline in physical or cognitive functions. Plus, she does not have abnormal oral structure and/or decline in multi-faceted oral function. Hence, this is a contrary case.

### Antecedents

Aging [31, 45], social frailty [46], and severe periodontitis [47] were possible influencing factors for oral frailty. Aging is an important factor for oral frailty [31, 45]. With aging, changes occur in the dentition, periodontium, oral mucosa, salivary gland function, and masticatory function. For instance, the teeth are darker in color, the number of teeth decreases, the periodontal tissues are reduced, the oral mucosa demonstrates a loss of elastic fibers, salivary flow is reduced, and the muscles of mastication is reduced. These declines in oral physiologic reserve increase vulnerability to stressors, thereby leading to increased oral frailty [38]. A study by Hironaka et al. indicated that social frailty might cause oral frailty [46]. It is probably because social frailty weakens interactions with others and decreases opportunities for conversation, leading to declined activities in muscles around the mouth and pharynx, slowed tongue movement, decreased masticatory performance, and reduced tongue pressure. This, in turn, increases the risk of developing oral frailty [46]. In addition, a cohort study design revealed that severe periodontitis increased the risk of oral frailty [47]. On the one hand, severe periodontitis causes tooth mobility or loss and decreases masticatory function, directly increasing the likelihood of onset to oral frailty [47]. On the other hand, severe periodontitis has indirect effects on oral frailty via social relationships [47]. A previous systematic review and meta-analysis indicated that periodontitis-related halitosis and degradation of aesthetics affect social relationships [48]. The associations between social relationships and oral frailty have also been revealed [49]. Therefore, severe periodontitis further affects oral frailty by social relationships.

### Consequences

**Fig. 2:**
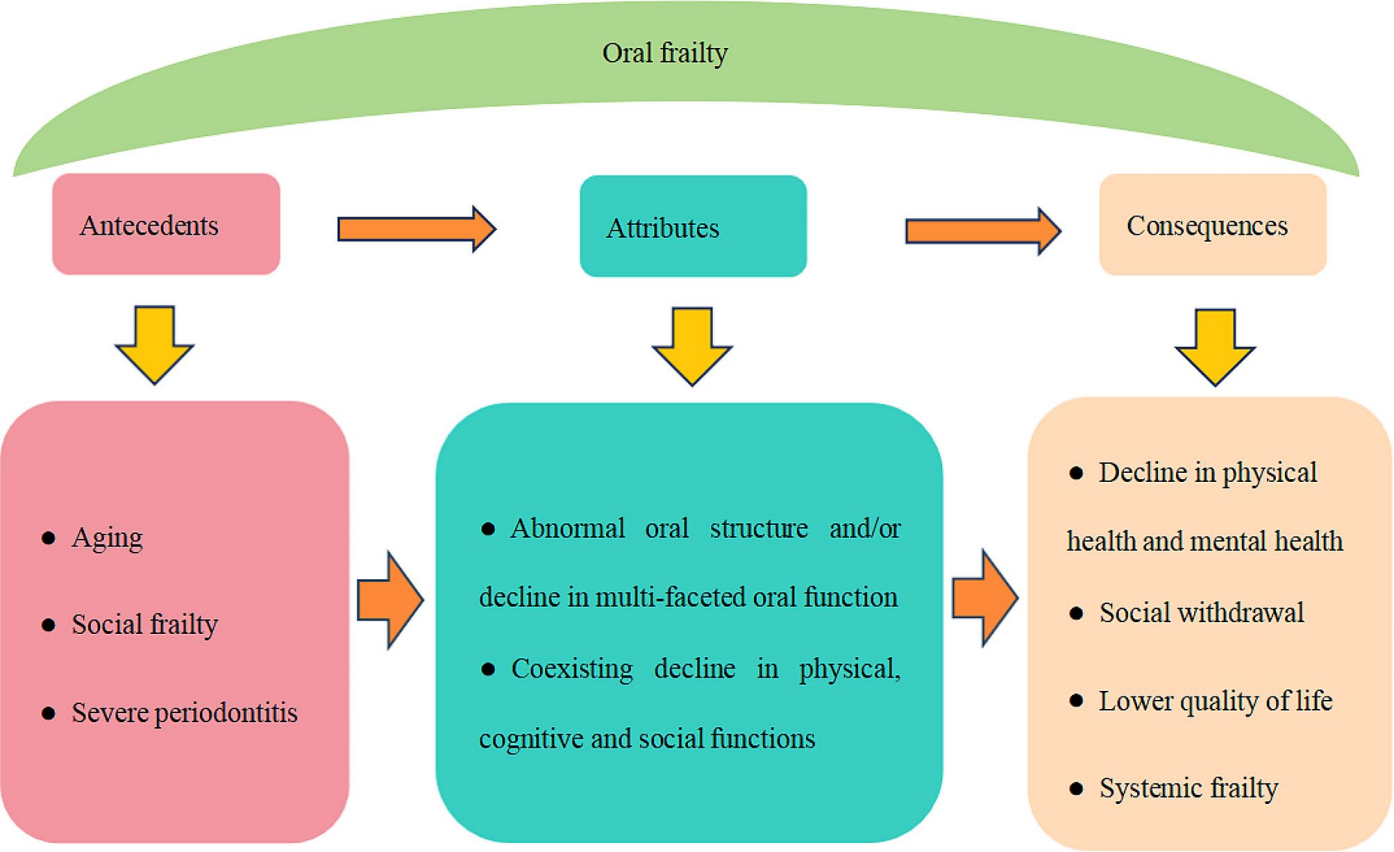
Antecedents, attributes, and consequences of oral frailty

Oral frailty could result in decline in physical health and mental health, social withdrawal [20], lower quality of life [50] and systemic frailty [19, 31]. The influence of oral frailty on physical health included decreased bone mineral density [51], pneumonia [22, 52], functional disability [50], physical frailty [31, 53], deteriorating nutritional status and increased drug administration [46, 54], and mortality [31], etc. As for mental health, oral frailty was a predictive factor for the incidence of mild cognitive impairment [55] and late-life depression in community-dwelling older adults [56]. The antecedents, attributes, and consequences of oral frailty are presented in Fig. 2.

### Empirical referents

The instruments for measuring oral frailty mainly include: the Kihon Checklist [57], the Oral Frailty Checklist/the Oral Frailty Index-8 (OFI-8) [7, 25], the Oral Frailty Five-item Checklist (OF-5) [26], Oral and Maxillofacial Frailty Index [58], and Oral Frailty Status [31].

The Kihon Checklist, developed by the Japanese Ministry of Health, Labor and Welfare, consisted of 25 questions in 7 categories: physical strength, nutrition, eating, socialization, memory, mood, and lifestyle [57]. A systematic review indicated that the Kihon Checklist is a reliable tool for predicting general frailty in older adults [59]. Hasegawa et al. selected three questions from the Kihon Checklist as items of oral frailty [20]. The three questions were respectively: “Do you find it difficult to eat hard foods compared to six months ago?”, “Do you choke on your tea or soup?”, and “Do you feel uncomfortable with your dry mouth?”. A score of 1 was assigned for a negative response to each of the question.

The Oral Frailty Checklist/the Oral Frailty Index-8 (OFI-8) was developed by the Japan Dental Association [7, 25], which consists of 8 items: (1) difficult to eat hard food, (2) choking, (3) using denture, (4) xerostomia, (5) less frequently going out, (6) feasible to chew hard food, (7) brushing teeth at least twice a day and (8) regular attendance of dental clinic. Item (1)~(3) scored 2 respectively, whereas other items scored 1 respectively. The maximum score was 11: low risk for 0–2 points, moderate risk for 3 points, and high risk for more than 4 points. The Cronbach’s α coefficient was 0.692 [60].

In order to assess oral frailty in various settings, not only for screening purposes, Tanaka et al. developed the Oral Frailty Five-item Checklist (OF-5) [26]. This instrument included five components: fewer teeth (frail response:<20 natural teeth), difficulty in chewing (frail response: yes), difficulty in swallowing (frail response: yes), dry mouth (frail response: yes) and low articulatory oral motor skills (oral diadochokinesis/ta/sound was < 6.0 times/s). The frail response of each item scored 1. The total score ≥ 2 indicated oral frailty.

Oral and Maxillofacial Frailty Index was developed by Choi et al., aiming to screening oral and maxillofacial frailty among older adults [58]. It included 5 items: difficulties in chewing, the necessity of water when eating dry food, difficulties in jaw or tongue movements, difficulties in speaking or pronunciation and difficulties in facial expression. Each item scored 1 (being never) ~ 4 (being often), and higher score indicated more severe oral and maxillofacial frailty. The Cronbach’s α coefficient was 0.704, and the retest reliability of each item was 0.70 ~ 1.00.

Tanaka et al. proposed that oral frailty was evaluated by Oral Frailty Status, including number of nature teeth, ODK/ta, tongue pressure, chewing ability, and subjective difficulties, and gave criteria for oral frailty cut-offs of each item [31]. For each item, 1 point was defined as a score below the cut-off point. The total score of 6 items: 0 points for non-oral frailty, 1–2 points for pre-oral frailty, 3 points and above for oral frailty.

## Discussion

Oral frailty means the abnormal oral structure and/or decline in multi-faceted oral function, accompanied by decline in physical, cognitive and social functions. Its antecedents were aging, social frailty, and severe periodontitis, whereas its consequences were decline in physical health and mental health, social withdrawal, lower quality of life and systemic frailty.

The explanations of “oral” and “frailty” in Cambridge Dictionary and Merriam-Webster Dictionary gives hints about the meaning of “oral frailty” that it is the weakness and worse health status or lack of strength of/related to oral cavity. This meaning reflects two dimensions of oral frailty: the oral cavity might have weak conditions and worse health status related to oral cavity exists.

As for the use of the concept, we reviewed the evolutions of oral frailty. The original definition “decline of oral function associated with aging” in 2013 stressed on the cause of oral frailty and only one dimension of it—the weak conditions regarding oral cavity [18]. This concept ignored the coexisting health conditions of declined oral function. Researchers mainly focused on the decline of oral physiological function such as chewing ability, difficulty in swallowing, slight choking, etc., till the Japan Dental Association proposed the definition of oral frailty [10]. The concept was explained from the perspectives of contents of oral function, cause, consequence, co-existing conditions and was a relatively comprehensive definition. However, with the continuous in-depth studies, researchers have gained better understanding of the antecedents and consequences of oral frailty, the specific manifestations of declined oral function and the accompanying symptoms. Our study provided new knowledge on the definition of oral frailty. Our definition of oral frailty consisted of two dimensions: abnormal oral structure and/or decline in multi-faceted oral function and coexisting decline in physical and cognitive functions. As for the former dimension, we added “abnormal oral structure” to the contents of oral frailty, as it appears repeatedly in the literature. However, the associations between the components of abnormal oral structure and oral frailty need to be further explored and tested. Our study indicated that coexisting decline in physical, cognitive and social functions was another attribute of oral frailty, which is similar to “decreased physical and mental reserve capacity” proposed by the Japan Dental Association. However, based on the current studies, we found that the coexisting decline manifested in three aspects—physical, cognitive and social aspects, which is more specific and comprehensive than that of the Japan Dental Association.

Antecedents were an important component for understanding a concept. However, the antecedents of oral frailty were less studied. Although aging has been stressed as a reason in many definitions of oral frailty, previous studies seldom examined the mechanism between aging and oral frailty. The outcomes of oral frailty were studied more than its antecedents, however, more evidence is needed.

Most studies investigating the association between oral frailty and physical function or nutritional status were cross-sectional, which could not make a causal conclusion [7, 36, 45]. Hence, the association between oral frailty and physical frailty needs to be further explored [43]. More evidence by causal design is needed to support the associations.

Currently, research on the development of assessment tools for oral frailty is limited. The predictive validity of tools for differentiating high and low risks for oral frailty has not been fully evaluated [61]. Although the Kihon Checklist was reliable for testing general frailty in older adults [59], the reliability and validity of the three items for oral frailty had not been tested. As for the Oral Frailty Checklist/the Oral Frailty Index-8 (OFI-8), Nomura et al. used Item Response Theory to analyze the characteristics of the instrument, and found the differentiating ability of item “brushing teeth at least twice a day” and item “regular attendance of dental clinic” low and these two items need to be modified [7]. The reliability and validity of the Oral Frailty Five-item Checklist (OF-5), which is a newly developed assessment tool for oral frailty, need to be tested. Oral and Maxillofacial Frailty Index is still in the development stage and has not been widely used. In our study, oral frailty has two dimensions, which will provide insight on the development of assessment tool from a different perspective.

There are some limitations in this study. Firstly, we conducted the literature search for studies published in English and Chinese only. This approach may have excluded relevant studies published in other languages. While the selected databases were deemed to have a wide range of literature sources, there is a possibility of omitting certain references. Including literature in other languages could provide additional insights into conceptual understanding. Secondly, quality assessment is not an integral part of a concept analysis. We did not perform quality assessments for the included studies; therefore, the results should be interpreted with caution. Finally, a concept analysis is largely interpretative by nature. There is a risk of subjectivity in analyzing definitions, and interpretation of oral frailty may vary among individuals, potentially introducing bias into the study.

## Conclusions

This study bridges the gap in previous studies on oral frailty by identifying its attributes, antecedents and consequences, cases, and empirical referents. The concept analysis analyzed its existing definitions and current studies and provided valuable insights for further research on developing scales to identify the status quo of oral frailty accurately and interventions to improve oral health for older adult populations. This concept analysis contributes to the correct use of oral frailty in regards to nursing theory, research, education, and practice.

### Electronic supplementary material

Below is the link to the electronic supplementary material.

Supplementary Material 1

## Data Availability

The datasets used and/or analyzed during the current study are available from the corresponding author on reasonable request.
